# Microsaccade Activity During Visuospatial Working Memory in Early-Stage Parkinson’s Disease

**DOI:** 10.3390/jemr18050046

**Published:** 2025-09-22

**Authors:** Katherine Farber, Linjing Jiang, Mario Michiels, Ignacio Obeso, Hoi-Chung Leung

**Affiliations:** 1 Integrative Neuroscience Program, Department of Psychology, Stony Brook University, Stony Brook, NY 11794, USA; 2 Department of Psychiatry and Behavioral Sciences, Stanford University, Stanford, CA 94305, USA; 3 Ph.D. Program in Neuroscience, Universidad Autónoma de Madrid, 28029 Madrid, Spain; 4 HM CINAC (Centro Integral de Neurociencias Abarca Campal), HM Hospitales, Móstoles, 28938 Madrid, Spain; 5 Cajal Neuroscience Centre, Consejo Superior de Investigaciones Científicas, 28002 Madrid, Spain; iobeso@csic.es

**Keywords:** microsaccades, region of interest, attention, eye tracking, Parkinson’s disease

## Abstract

Fixational saccadic eye movements (microsaccades) have been associated with cognitive processes, especially in tasks requiring spatial attention and memory. Alterations in oculomotor and cognitive control are commonly observed in Parkinson’s disease (PD), though it is unclear to what extent microsaccade activity is affected. We acquired eye movement data from sixteen participants with early-stage PD and thirteen older healthy controls to examine the effects of dopamine modulation on microsaccade activity during the delay period of a spatial working memory task. Some microsaccade characteristics, like amplitude and duration, were moderately larger in the PD participants when they were “on” their dopaminergic medication than healthy controls, or when they were “off” medication, while PD participants exhibited microsaccades with a linear amplitude–velocity relationship comparable to controls. Both groups showed similar microsaccade rate patterns across task events, with most participants showing a horizontal bias in microsaccade direction during the delay period regardless of the remembered target location. Overall, our data suggest minimal involvement of microsaccades during visuospatial working memory maintenance under conditions without explicit attentional cues in both subject groups. However, moderate effects of PD-related dopamine deficiency were observed for microsaccade size during working memory maintenance.

## 1. Introduction

Visuospatial working memory refers to the active maintenance and processing of object locations in the visual environment around a person [1], with spatial attention often considered as the underlying rehearsal mechanism [2,3]. The oculomotor system has long been associated with cognitive processes including spatial attention and working memory, though the relationship and interaction between these systems remain unclear (see reviews [4,5]). Microsaccades, the tiny eye movements that occur during gaze maintenance [6,7], are thought to monitor environmental events in greater detail for further processing and to prevent visual fading during fixation [8]. Microsaccades exhibit similar oculomotor properties as the larger saccades [9], suggesting a microsaccade–saccade continuum in oculomotor control with potentially shared underlying neural substrates [10,11,12,13]. While there is a significant body of research linking microsaccades and attentional processes, accumulating evidence suggests a role relating to internal active maintenance of memory beyond simply responding to external cues (see review [14]).

Among the brain structures involved in oculomotor control, the basal ganglia are postulated to play a critical role for triggering saccadic eye movements [15,16] (see Figure 3 in [16] for a schematic representation). Dopamine, a major modulatory neurotransmitter that is implicated in goal-directed behaviors, is involved in mediating the basal ganglia output to the superior colliculus [17,18,19]. This mediation is postulated to regulate the interaction between the superior colliculus and the brainstem omnipause and premotor burst neurons, switching between opposite firing and non-firing states for the two types of cells [20] that determine the generation (burst neuron firing) and termination (omnipause neuron firing) of a saccade [21,22]. Dopamine is also implicated in a multitude of cognitive functions [23,24], thus linking the oculomotor and cognitive systems. Indeed, dopamine depletion is shown in animal models to cause deficits in voluntary saccade control and working memory during goal-directed behaviors [25,26].

Parkinson’s disease (PD) is characterized by the loss of dopaminergic cells in the substantia nigra, which has a major impact on basal ganglia-mediated cognitive and oculomotor functions [27,28]. Dopamine loss results in motor, including oculomotor (reviewed in [29]), and cognitive dysfunctions, with visuospatial working memory particularly impaired in PD [30,31]. Additionally, recent studies of individuals with early to moderate PD showed a general trend of increased oculomotor instability and amplitude, velocity, and latency variability in saccades [32,33,34] and in microsaccades [35,36]. This trend seems to be caused by overall impaired inhibition of spontaneous or reflexive eye movements in people with PD [37,38]. A non-linear effect of dopaminergic medication, such as L-DOPA, has been previously proposed, where both too little and too much dopamine lead to oculomotor [39] and cognitive [40] dysfunctions. Although numerous studies examined saccades and working memory in PD, there have not yet been studies focusing on fixational microsaccades during working memory maintenance. Thus, it is not clear whether microsaccades play any significant role in visuospatial working memory dysfunction in PD.

Eye tracking as a tool has informed the study of cognitive processes such as selective spatial attention [41,42] and memory processes [43,44,45] during both experimental tasks [46] and complex natural tasks [47,48]. More specifically, previous studies of healthy young adults showed that microsaccades are modulated by memory cues and shifts in covert attention, suggesting a role of microsaccades in supporting mnemonic processes [48,49,50,51,52,53]. Some studies have also linked microsaccadic temporal activity to both visual and auditory anticipation [54,55]. It has been suggested that spatial cueing transiently activates the superior colliculus, prompting the generation of microsaccades biased towards the target [56]. Working memory demand also modulates microsaccade activity and produces a characteristic pattern of decreases and increases in rate, the amplitude of which can also be affected by memory load [51,52,57,58,59]. A number of studies have also reported a relationship between microsaccade directions and visual working memory [51,52,60]. However, null effects of stimulus or cue location on microsaccadic direction were reported for similar task conditions [36,61]. Thus, it is still not clear whether the attentional cue or working memory demand itself may modulate microsaccade properties. Studying the link between microsaccade properties and spatial working memory in PD would help clarify the potential underlying cognitive mechanisms. Such knowledge may allow for better probing into how attention, learning, and memory strategies are applied in complex tasks by tracking eye movements [62,63].

The goal of the current study was to examine the relationship between dopamine deficiency in PD and microsaccade activity during spatial working memory maintenance. We monitored and recorded the eye positions of a group of early-stage PD and healthy older individuals while they performed a memory-guided saccade task. We chose to use this simpler paradigm to investigate whether microsaccade activity is altered in early-stage PD during working memory maintenance prior to memory-guided saccades. We hypothesized that microsaccades play a functional role in spatial working memory and expected them to show distinct responses to the variable target locations. We further hypothesized that microsaccadic activity during spatial working memory maintenance would be impaired in PD and explored whether it is modulated by dopaminergic medication status.

## 2. Materials and Methods

### 2.1. Participants

Informed consent was obtained from all subjects involved in this study. Sixteen individuals (5 female) diagnosed with early-stage Parkinson’s disease (PD) (Hoehn and Yahr score of 1 or 2) [64] from the Stony Brook community (*n* = 11) and Hospitales Madrid (*n* = 5), with an average age of 61.06 years (SD = 9.77, range of 43–77 years), participated and were included in the final analysis (Table 1). PD participants in Madrid did not differ in their absolute error of task performance from Stony Brook participants (*p*’s > 0.1), and their main sequences did not differ significantly (*p* > 0.5). Four other participants were excluded due to a high percentage of eye-tracking artifacts overall, and one was removed due to an atypical microsaccade main sequence, in addition to the artifacts. Motor symptoms and cognitive symptoms were assessed using the UPDRS III [65] and MoCA [66] assessments on the experiment days. Participants with PD performed the task twice, coming in on two occasions separated by approximately 2 weeks. In one visit they were asked to stop taking their medication 12 h prior (PD OFF), while on their other visit they were taking their medication as clinically advised (PD ON). The order of the medication status on visits was counterbalanced across patients.

Thirteen additional healthy individuals (7 female) from the Stony Brook community with an average age of 69.15 years (SD = 9.09, range of 48 to 81 years) served as a control group (OHC). One other participant was excluded from this group due to a high percentage of eye-tracking artifacts.

### 2.2. Apparatus and Visual Stimuli

Participants performed the task in a dimly lit room, sitting with their head stabilized by a chin rest 70 cm from a computer monitor (screen size 37.5 cm × 30.2 cm, 1024 × 768 pixels). Five of the participants (data collected in Spain) sat 60 cm from the monitor (screen size 54 cm × 30 cm, 1920 × 1080 pixels). Even with this variability, individual subject data from the two sites were comparable. Monocular eye movements were recorded using a desktop mounted infrared video-based eye-tracker (Stony Brook: Eyelink 1000; Madrid: Eyelink 1000 Plus, SR Research, Ottawa, ON, Canada) at 1000 Hz. We performed 9-point calibration and validation at the beginning of every other task block to make sure eye positions were correctly registered during task performance. All participants successfully passed calibration and validation checks (average fixation error from dots < 0.5°, max error < 1°).

**Figure 1:**
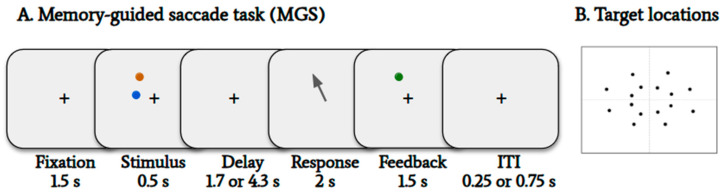
The memory-guided saccade task. (**A**) The schematic diagram shows the sequence of task events in a trial. An instruction was shown at the beginning of each block of trials indicating which color is the target, while the other color is to be ignored. (**B**) The 16 possible target and distractor dot locations.

Experimental Builder software (version 2.3.38, SR Research, Ottawa, ON, Canada) was used to present the visual stimuli. A black fixation cross subtending 0.8° of visual angle was shown in the center of the screen during the task. Targets and distractors were presented as blue and orange dots of 1° of visual angle, with the target dot reappearing as green for feedback at the end of each trial. There were 4 possible dot eccentricities (3°, 5°, 7°, 9° of visual angle) and 8 possible polar angles (from 22.5° to 337.5°, in 45° increments), with a total of 16 possible dot locations (Figure 1B). Jittering was applied to the dot eccentricities (up to 0.5° of visual angle) and dot angles (up to 1°). Target-and-distractor pairs were placed in the same quadrant at different eccentricities and at either the same or different visual angles (approximately 45° apart). The different combinations of target–distractor relationships were counterbalanced across trials.

### 2.3. Visuospatial Working Memory Paradigm

We used a memory-guided saccade task (Figure 1A). On each trial, after an initial fixation of 1.5 s, a target and distractor stimulus was presented for 0.5 s. This was followed by a variable delay interval, either 1.7 or 4.3 s long. The participants were instructed to shift their gaze to the remembered location of the target dot when the fixation cross disappeared at the end of the delay period. Two seconds after fixation cross disappearance, a feedback stimulus (green dot) was presented in the original target location, and the participants were asked to shift their gaze to this dot location before looking back at the center fixation cross. Participants were instructed to maintain their fixation on the central cross for the entire trial, except when generating the saccade to the target at the end of the delay. There was a variable inter-trial interval of either 250 or 750 milliseconds between trials.

The study session consisted of 1 practice block (16 trials) and 4 task blocks with 32 trials per block, resulting in 144 trials. At the beginning of each block of trials, an instruction was presented to indicate which color stimulus (blue or orange) was the target and the other color dot was always irrelevant. The target and distractor color varied alternatively across runs. The target and distractor locations of each trial within a block were pseudo-randomly selected from the 16 possible locations, and the delay duration was pseudo-randomly selected from the 2 possible intervals. Each possible target location was only repeated once within a block, and never repeated (either by eccentricity or angle) in consecutive trials.

### 2.4. Microsaccade Identification and Analysis

Horizontal and vertical (pixel) eye-position data was imported into MATLAB (2019a) for conversion and processing using custom-written MATLAB scripts adapted from Adrian Etter and Marc Biedermann (Edf2Mat) and our own lab. All saccadic and microsaccadic eye movements were first identified with a velocity-based detection code, consistent with methods used in the literature [48,49]. Detection was based on predefined minimums of velocity (23°/s), acceleration (5000°/s^2^), amplitude (0.2°), and duration (8 ms). Parameters were selected based primarily on Otero-Millan and colleagues’ clustering AI method [67] and were comparable to common ranges in the literature [36,49]. Microsaccades were then identified and selected based on amplitude (0.2–1°). Preliminary analysis showed that 70–77% of all saccadic eye movements made during initial fixation, stimulus presentation, and delay periods were under 1° of amplitude (see Supplementary Figure S1 for detailed group average breakdowns of saccade amplitude distributions).

Time points with blinks, missing data, outside-of-screen data, and abnormal pupil size and speed were removed, with blink detection following the default Eyelink detection method with an added 100 ms time window extension. If the participant broke fixation during the delay period and then moved their eyes to a new location, microsaccades in the new location were excluded from analysis. For this detection, an average location and measure of variability (standard deviation) of all individual microsaccades was calculated. Microsaccades that were more than 2.5 SD away from the average were considered erroneous and excluded (out of total eye movements: OHC: 5.6–24.5%; PD OFF: 5–43.2%, PD ON: 6.9–51.1%). Additionally, square wave jerks (SWJs), which are pairs of microsaccades that are in approximately opposite directions, of approximately equal sizes, and have a short inter-saccadic interval [68,69], were also identified and excluded (Table 1) based on parameters approximated from reported SWJ ranges described in [70] (directional dissimilarity index of >135°, magnitude similarity index of ≥0.32, and intersaccadic interval of ≤310 ms). Exclusion of SWJs was not shown to affect the distributions and activity of microsaccadic characteristics [48]. It was shown in the same work that SWJs are primarily directed horizontally, so they were excluded (OHC: 22.8–64.6%; PD OFF: 6.1–62.2%; PD ON: 13.1–55.8%) to increase sensitivity in target location-bias analyses.

Microsaccades were then analyzed for two task epochs, with the initial fixation period serving as the control (period with no memory demand) for analyses, and the delay period served as the main period of interest, since working memory was assumed to be actively maintained before the memory-guided saccadic response. Data was initially analyzed separately for the two delay duration trials (1.7 s and 4.3 s) (see Supplementary Materials), and since no differences in eye movement behavior were found for the two delay durations (1.7 s and 4.3 s), data from both delay durations were combined in the final analyses and figures reported here.

For each individual, their basic microsaccade characteristics including number, amplitude, velocity, duration, and rate were calculated for each task epoch. The strength of the “main-sequence” relationship between saccade amplitude and velocity was measured by calculating the Spearman-rho rank correlation coefficient of these two variables (since it is less sensitive to outliers than the Pearson linear correlation). Given the potential presence of microsaccades with unrealistic velocities, and our minimal outlier removal, we also performed a linear regression between amplitude and peak velocity for each subject using Matlab’s robustfit function, and used the adjusted velocity values to compare average velocity between groups.

The timecourse of microsaccade rate was constructed using a sliding window of 100 ms in 1 ms steps and calculated in Hz. The rate at each time point was adjusted by each individual’s baseline rate, which was averaged from a stable window (600–800 ms) during the initial fixation period. Microsaccade rate during working memory was extracted from three windows during the delay period: early delay (0–500 ms), middle delay (600–1100 ms), and late delay (1200–1700 ms for short-delay trials; 3800–4300 ms for long-delay trials).

### 2.5. Statistical Analysis

Microsaccades of both delay trial types were combined for the final analyses. Two-way mixed ANOVAs were conducted to test for the effects of disease state (OHC vs. PD OFF, OHC vs. PD ON) and task epoch (initial fixation vs. delay) on basic microsaccade characteristics (amplitude, adjusted velocity, duration, acceleration). Two-way repeated-measures ANOVAs were conducted to test for the effect of medication (PD ON vs. PD OFF) on these basic microsaccade characteristics. The assumption of variance homogeneity was evaluated using Levene’s test.

Repeated measure t-tests were performed to determine the main effects of target side (left vs. right visual field) during the delay period on microsaccade directional bias. Microsaccade directional bias was estimated by measuring laterality index, with (Number_right-Number_left) divided by (Number_left + Number_right), where microsaccades directed to the right side were assigned a positive sign, and microsaccades to the left side were assigned a negative sign. For a second, more nuanced directional bias analysis during the delay period, we calculated the angular difference between each microsaccade (endpoint x,y-start x,y vector) and its corresponding trial’s target location (target x,y-fixation cross x,y vector). Separate angular-difference distributions were also produced for the first, middle, and last 500 ms of the delay. For comparison, we also calculated the angular differences in the microsaccades during initial fixation from the same target locations. Given that we were using group-level distributions for our statistics, we used non-parametric tests. Within-group, we tested for temporal differences across the delay time bins (Friedman’s tests) and between the initial fixation and the whole delay (Wilcoxon’s signed rank). Wilcoxon’s rank sum tests were performed to assess group differences in delay distributions (OHC vs. PD OFF, OHC vs. PD ON).

We also assessed the possible effects of target eccentricity on microsaccade amplitudes (per-group repeated measures ANOVAs) (see Supplementary Materials). Independent measure and repeated measure *t* tests were also used to compare groups’ main sequence Spearman’s rho rank correlation coefficients.

All post hoc *t* tests were controlled for multiple comparisons (Bonferroni-corrected) and Cohen’s d was calculated for significant results.

Group differences in rate activity pattern were analyzed for the initial period and three 500 ms bins of the delay period. Average rates were compared between groups for the initial fixation/delay epoch time windows (early delay: 0–500 ms in delay, middle delay: 600–1100 ms, late delay: 1200–1700 ms (for short-delay trials)/3800–4300 ms (for long-delay trials)) using 2-way mixed effects (OHC vs. PD ON, OHC vs. PD OFF) and repeated measures ANOVAs (PD ON vs. PD OFF). The main effects of time and group (or medication status) were assessed, and *p* values had Greenhouse–Geisser corrections applied when sphericity was not maintained. Since this approach does not consider the temporal pattern of rate within each of the time bins, we also sampled the rate at every 50 ms within them, and performed 2-way ANOVAs (Group × Time), to more closely assess rate differences within each time bin.

## 3. Results

**Figure 2:**
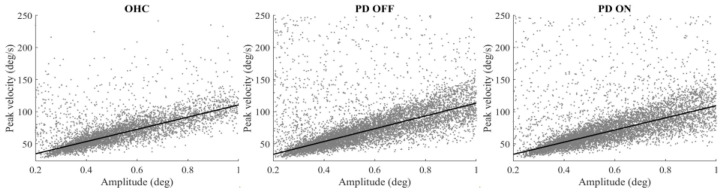
Main sequence of microsaccades for healthy controls (OHC) and PD individuals “on” (PD ON) and “off” (PD OFF) dopaminergic medication. The linear regression lines fitted on the group level using MATLAB’s robustfit are shown for visualization.

We first examined the main sequence relationship between microsaccade amplitude and velocity to confirm whether the PD and OHC participants have comparable basic saccade mechanisms. Spearman’s rank correlation confirmed that the microsaccades of the OHC subjects during the working memory task show a moderate linear relationship between the microsaccade amplitude and velocity (0.35 < r_s_ < 0.89, *p*’s < 0.001). The PD participants, both “on” and “off” their medication, also exhibited microsaccades of a similar linear main sequence relationship (PD OFF: 0.25 < r_s_ < 0.87, *p*’s < 0.001; PD ON: 0.44 < r_s_ < 0.87, *p*’s < 0.001) (Figure 2). While the main sequence relationship seems slightly weaker in the PD group, it was not statistically different from the healthy controls (*p*’s > 0.6) and not by medication status (*p* > 0.5). There was no significant change in main sequence relationship from initial fixation to delay period for either OHC or PD (paired *t* tests, *p*’s > =0.1). Main sequence linearity was also not significantly correlated with individual differences in motor symptom severity (UPDRS III total score) or general cognitive ability (MoCA) across the PD subjects (−0.48 < r < 0.15, *p*’s ≥ 0.06).

**Figure 3:**
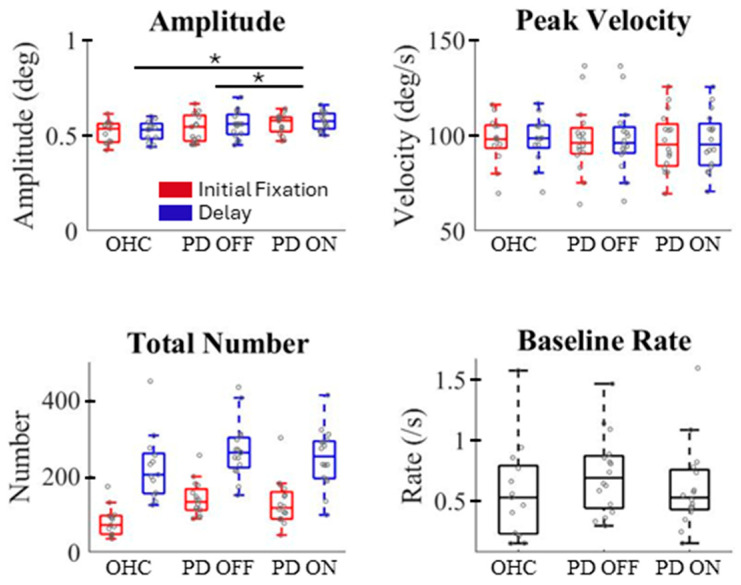
Comparing basic characteristics of microsaccades between healthy controls (OHC) and participants with PD, “off” (PD OFF) and “on” (PD ON) dopaminergic medication. Amplitude is the average size of microsaccades during the initial fixation and delay periods of the task. Peak velocity was estimated from robustfit. Number is the total number of microsaccades during each epoch. Baseline rate was calculated from the 600–800 ms time in the initial fixation. Red boxes represent initial fixation data, and blue boxes represent delay period data. Boxplots’ central mark indicates the median, edges indicate the 25th and 75th percentiles, and dashed lines extend to the most extreme data points not considered outliers. Individuals’ data points are shown as scatters on the boxplot. * *p* < 0.05.

The PD participants made similar numbers of microsaccades compared to healthy controls during each task epoch (Figure 3, lower left). However, the PD participants appeared to generate slightly larger microsaccades relative to healthy controls (Figure 3, top left), with two-way ANOVAs revealing a significant main group effect for PD ON vs. OHC (*F*(1,27) = 6.06, *p* = 0.02) but not PD OFF vs. OHC (*F*(1,27) = 1.28, *p* = 0.27), and no significant main effect of epoch (*F*’s < 1) or group by epoch interaction (PD ON vs. OHC: *F*(1,27) = 1.03, *p* = 0.32; PD OFF vs. OHC: *F*(1,27) = 2.23, *p* = 0.15). The amplitude difference between PD ON and OHC was driven by the delay period (*t*(15) = 2.79, *p* = 0.001, d = 1.03). In addition, the two-way repeated measures ANOVA showed both a significant medication effect (PD ON vs. OFF: *F*(1,15) = 6.49, *p* = 0.02) and a significant epoch effect (delay vs. initial fixation *F*(1,15) = 15.44, *p* = 0.001) on microsaccade amplitude, but not their interaction (*F* < 1), with the PD OFF’s epoch amplitude difference surviving multiple comparisons of the post hoc t-tests (Bonferroni-adjusted alpha = 0.025; PD ON: *t*(15) = 2.28, *p* = 0.038, d = 0.20; PD OFF: *t*(15) = 3.09, *p* = 0.007, d = 0.29). Not surprisingly, microsaccade duration was slightly longer for the PD participants (PD ON vs. OHC: *F*(1,27) = 7.34, *p* = 0.01; PD OFF vs. OHC: *F*(1,27) = 3.31, *p* = 0.084). No other main microsaccade characteristics (e.g., velocity, baseline rate) showed any main effects of disease group/medication, task epoch, or their interactions (*p*’s > 0.05). Since the PD and OHC cohorts included in the final analysis differed in age, we used an ANCOVA test to further confirm that the group differences in microsaccade amplitude and duration remained significant with age as a covariate. Taken together, the PD individuals produced slightly larger microsaccades than the OHC, particularly during working memory maintenance.

**Figure 4:**
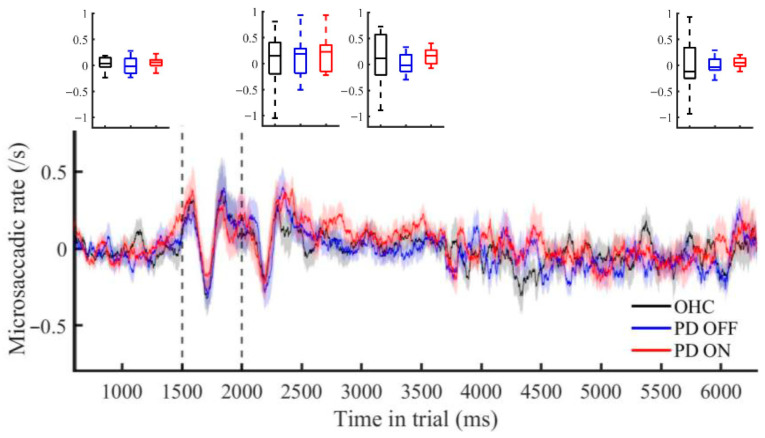
Microsaccade rate changes over time during the memory-guided saccade task. Changes in the microsaccade rate, adjusted by the baseline rate, are shown for the PD group on (red)/off (blue) medication relative to the healthy old adults (OHC) (black). Shaded areas represent standard error. The first and second dashed lines, respectively, represent stimulus onset and stimulus offset (which is also delay onset). Initial fixation is the period from the y-axis to the first dashed line, and the delay period runs from the second dashed line to the end. Above the rate diagram shows box plots of averaged rate during four selected time bins (entire initial fixation and 500 ms delay bins). See Supplementary Figure S2 for separate timecourses for short-delay trials vs. long-delay trials.

The microsaccade rate variation during the delay period showed a similar pattern for the two subject groups and PD medication status, consisting of an initial dip in rate approximately 0–200 ms after the stimulus offset, followed by a peak during approximately the next 200 ms before returning to baseline (Figure 4). This pattern was also apparent during the stimulus presentation. For separate timecourses for short-delay trials and long-delay trials, see Supplementary Figure S2.

We used two-way mixed effects ANOVAs to test whether microsaccade rate varied across time (the initial fixation and early, middle, and late delay) differently in PD participants relative to the controls. There were no significant group-by-time interactions (*F*’s < 1), and the repeated measures ANOVA also showed no statistically significant medication effect on microsaccade rate over time (*F* < 1). We further examined the first and last 500 ms of the delay in greater temporal resolution as these windows of time have the most variability and potential anticipatory effects towards the end of the delay. These additional mixed effects ANOVAs showed no significant main effects of group (*F*’s < 1) or group-by-time interactions in either time bin (early delay: OHC vs. PD OFF: *F*(9,243) = 1.40, *p* = 0.19; OHC vs. PD ON: *F* < 1; late delay: *F*’s < 1). However, the repeated ANOVA for PD ON vs. PD OFF showed a significant main effect of time for both early delay (*F*(9,135) = 6.9, *p* < 0.001) and late delay (*F*(9,135) = 3.8, *p* < 0.01), but not for medication-by-time interaction (early delay: *F*(9,135) = 1.55, *p* = 0.20; late delay: *F* < 1), suggesting that PD participants, regardless of medication status, exhibit similar fluctuations in microsaccade rate over time.

**Figure 5:**
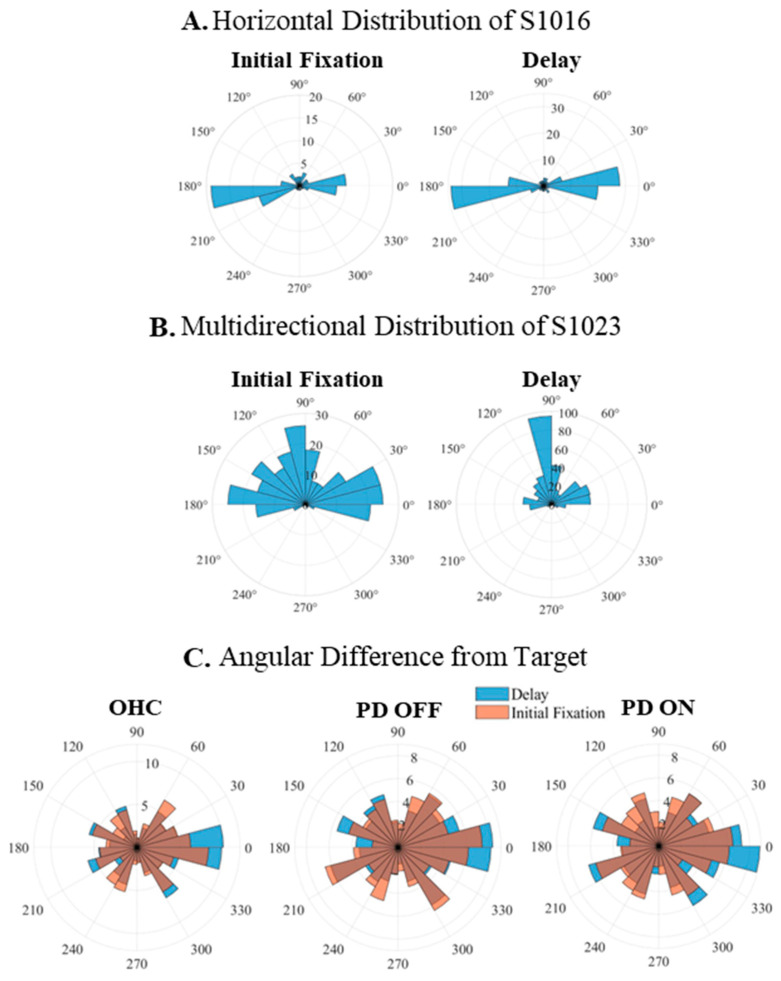
Microsaccade directional preference. The first two rows show the two common patterns of microsaccade directions during the initial fixation and delay periods of the memory-guided saccade task for representative participants (**A**,**B**). Most participants (15/16 OHC, 14/16 PD OFF, 12/16 PD ON) generated (**A**) horizontally oriented microsaccades, while some showed (**B**) multidirectional microsaccades (1/16 OHC, 2/16 PD OFF, 4/16 PD ON). (**C**) Group-level angular difference distributions (microsaccade vs. target direction) for initial fixation (orange) and delay period (blue) microsaccades in the OHC and PD participants. See Supplementary Figure S4 for angular difference distributions for early vs. late delay period microsaccades.

Lastly, we examined whether the directional preference of microsaccades varied by disease and medication status. The majority of participants (15/16 OHC, 14/16 PD OFF, 12/16 PD ON) showed mostly horizontally directed microsaccades (i.e., >60% of microsaccades within 60 degrees of the horizontal axis, although many participants had >80% in this range) during both initial fixation and delay periods, while others (1/16 OHC, 2/16 PD OFF, 4/16 PD ON) showed a multidirectional pattern (Figure 5A,B). To note, the PD participants who showed a multidirectional pattern when on medication were not the same participants as off medication. By calculating the laterality index, we further found that each group of participants showed similar left/right hemifield preferences during the delay period, regardless of the left/right target locations (OHC: *t*(12) = 1.40, *p* = 0.20; PD OFF: *t*(15) = 0.95, *p* = 0.36; PD ON: *t*(15) = 0.63, *p* = 0.54). To further test for directional bias in microsaccades, we calculated their angular difference from the target (Figure 5C). Although visually there seems to be differences in angular distribution, there were no significant epoch (Wilcoxon’s signed rank test) or temporal (Friedman’s test) effects on each group’s distribution (*p*’s > 0.6), and no significant group differences for the delay period distribution by Wilcoxon’s rank sum test (*p*’s > 0.7).

## 4. Discussion

While a few previous studies have noted alterations in PD participants’ microsaccade characteristics during fixation and voluntary saccade tasks [36,71], our findings extended this to a spatial working memory task. Our PD participants showed modest group-level differences in microsaccade basic characteristics (amplitude and duration) in comparison to the older healthy controls, with PD individuals “on” medication showing slightly larger microsaccades than “off” medication. However, both groups of participants in this experiment showed primarily horizontally directed microsaccades, as some previous studies have also reported [8,36], and no clear biases in direction to the targets during working memory maintenance. Importantly, the slight alterations in microsaccade parameters (amplitude and duration) do not seem to be symptom-related, as they were not associated with individual differences in motor symptom severity or general cognitive ability across the PD subjects. Perhaps the microsaccade changes found for our PD subjects are related to downstream brainstem effects or an interaction between superior colliculus and brainstem control of spontaneous saccades, rather than goal-directed saccades.

### 4.1. Dopamine Deficiency Alters Microsaccade Characteristics and Activity

Our findings show alterations in microsaccade amplitude and duration in a group of early-stage PD participants in comparison to a group of controls. However, the lack of statistically significant differences in directional bias between the PD and control groups does not suggest that the slightly larger microsaccades were generated with a specific purpose for spatial working memory maintenance. Importantly, the microsaccades generated by these PD participants demonstrated a comparable main sequence to those generated by the older healthy participants. While some previous studies have reported main sequence differences in PD participants compared to healthy controls [36,72], others did not find this difference [33,69]. Perhaps these data reflect differences between large saccades and the microsaccades we examined, or parameters such as disease progression, given that our participants were all in early stages of the disease. This thus suggests that microsaccades may weakly reflect the general oculomotor deficits and variability that are consistently observed in studies with PD participants [33,69].

Previous studies have shown that levodopa medication increases saccadic accuracy and improves smooth pursuit, indicating improved oculomotor function [73,74]. The limited medication effects on microsaccade characteristics and activity in our study do not strongly support medication-related improvement extending to these small eye movements. In contrast, our PD participants on medication exhibited greater difference in amplitude size from healthy controls compared to those off medication. This could potentially be attributed to the inverted-U hypothesis of dopamine function, whereas participants’ levodopa medications in early stages of the disease may increase their dopamine levels beyond ideal levels for these particular oculomotor characteristics [40]. However, further studies and replication are needed to determine whether these medication effects are effective early biomarkers of visuospatial dysfunction and dopaminergic imbalance. These findings further suggest that while dopamine replacement may compensate for larger, intentional, or goal-directed saccades, it apparently does not help keeping fixation steady [75], which is a potential non-dopaminergic underlying source of disrupted oculomotor system’s control of fixation. Indeed, other clinical treatments for people with PD, such as high-frequency subthalamic nucleus stimulation, have been suggested to improve microsaccade performance in memory-related tasks [76,77]. Future studies should directly compare the effects of these treatment outcomes on microsaccades.

### 4.2. Microsaccade Rate During Working Memory

The rate pattern of microsaccade activity in the delay period (a decrease in rate followed by an increase in rate) suggests that some reactionary behavior occurs compared to the baseline period, where no systematic variation in microsaccade rate occurred. Similar patterns of a decrease and increase in rate have been noted in several previous studies of healthy and PD participants [36,51,52,58,78].

The observation of a similar rate pattern in the stimulus presentation period may suggest that microsaccades are involved in both working memory retention as well as stimulus perception and encoding. These functions are not mutually exclusive, so microsaccades may reflect both functions. The rate pattern may also be a reflection of attention mechanisms related to a change in visual information processing (i.e., stimulus appearance and disappearance) rather than the working memory demand itself. Further studies could use variable working memory difficulty load (similar to [58], although this particular study used young healthy participants), to elucidate possible differences in the activity for the rate changes related to attentional changes versus working memory maintenance. Dalmaso and colleagues [58] associated this pattern with variable working memory loads in a retention period. However, they did not show rate activity in their ‘learning frame’ when stimuli first appeared. In other reports, a rate activity pattern similar to ours in both stimulus onset and offset periods is also noticeable ([78], Figure 4, p. 7). Additionally, this rate pattern is seen during stimulus presentation in previous studies without a delay period [61].

### 4.3. Lack of Directionally Biased Microsaccades

While previous studies of healthy young adults postulated a role for microsaccades during working memory due to their directional bias towards the to-be-remembered target [48,49,51,52], we did not find any significant directional bias in the current study. Most participants, PD or control, in the current study showed microsaccades that were mostly horizontally directed, regardless of target location or task event during the memory-guided saccade task. This is consistent with studies that have observed both task- and target-irrelevant microsaccade activity patterns. For example, Tse and colleagues [79] reported rightward directed microsaccades regardless of cue location, and several studies have observed a preference for horizontally directed microsaccades similar to ours [8,36,48,51]. One possible explanation for this difference is the absence of an attentional cue during the working memory period in the studies that do not find directional biases (like ours) compared to those which do [49,80,81,82].

Studies which have found target-biased microsaccades often involved additional demands on attention or memory. Specifically, several recent studies that found significant directional effects on microsaccades [51,52,53,60] differed in the timing and difficulty of the task compared to our study. Our participants were pre-cued as to which stimulus dot was the target, and thus they could ignore the distractor, while the studies cited above used a retro-cue task, so their participants would need to select one stimulus out of two (or more) remembered items during the delay. Thus, additional memory selection (or attentional bias) operation might take place during working memory delay. Separately, for these same studies the two potential targets were both located along one axis (horizontal, vertical, or diagonal), which could have encouraged participants to look along this axis and produce a significant directional bias. Furthermore, as we discuss in our Limitations Section, these studies also had much more data from a larger number of trials. It is possible that the combination of a task with less selection demand, un-aligned target locations, and fewer data did not provide us with sufficient conditions or power to see weak directional effects. For a task focused more purely on visuospatial working memory like ours, the lack of directional bias suggests that microsaccades are not always used to covertly assist in remembering target location. The results of this study argue against the utility of microsaccade directionality as a general function of internal working memory maintenance, regardless of age or dopamine medication status.

To clarify the relationship between microsaccades and spatial attention versus working memory, future studies should directly compare pure working memory maintenance (similar to this study) and working memory with a spatial cue such as [48] or aligned target locations [51]. Further, future studies with a larger sample size can use a combination of eye tracking and cognitive task design to investigate attention and working memory deficits in PD or other clinical patient groups, as well as in applied sciences and naturalistic conditions [63].

### 4.4. Limitations

One limitation of our study was the small sample size (16 PD and 13 OHC), which was partially due to data collection occurring through the later part of the COVID-19 pandemic. This small number could have contributed to the overall noise in the signal of the characteristics and activity, due to lack of statistical power. Although previous studies have observed increased oculomotor variability in people with PD [33,34,72], only small medication effects were revealed at the group level. Even for general findings, such as the lack of directional biasing, the results could perhaps be due to our limited sample size. It is possible that the absence of retro-cueing or alignment of target locations in combination with our small sample size may have prevented the detection of weak directional biases or medication effects. It is also possible that untested non-dopaminergic mechanisms, such as those involving the subthalamic nucleus, may instead be involved in PD oculomotor deficits or the lack thereof in our study [83].

Taken together, our data suggest rather limited involvement of microsaccades in correspondence to visuospatial working memory demand under conditions without any explicit attentional cue. The results also suggest moderate effects of PD-related dopamine deficiency on microsaccadic activity during a spatial working memory task.

## Figures and Tables

**Table 1:** Demographics.

Title 1	OHC (*n* = 13) Mean (SD)	PD (*n* = 16) Mean (SD)	*p* (OHC/PD)
Age	69.15 (9.09)	61.06 (9.77)	0.03
Sex (f/m)	7/6	7/9	0.22
Education	15.15 (3.13)	16.9 (2.92) ^a^	0.18
MoCA	26.92 (1.38)	(OFF) 26.5 (2.12) ^a^	0.87
		(ON) 26.82 (2.14) ^b^	0.57
HY		1.73 (0.47) ^b^	
LEDD		632.25 (455.78) ^c^	
UPDRS		(OFF) 28.5 (5.83)	
		(ON) 22.2 (6.46) ^d^	
Absolute error	1.56 (0.30)	(OFF) 1.82 (0.52)	0.04
		(ON) 1.83 (0.43)	0.02
Trials included *	114 (9.68)	(OFF) 120.31 (7.02)	0.05
		(ON) 117.31 (12.91)	0.45
SWJ (%)	41.15 (10.74)	(OFF) 38.08 (12.52)	0.50
Broken fixation (%)	14 (5.5)	(ON) 36.4 (14.92)	0.34
		(OFF) 18.2 (10.34)	0.20
		(ON) 21 (11.93)	0.06

Older healthy control group (OHC) and participants with Parkinson’s disease (PD). Spatial working memory performance is measured as primary saccade error from target location in degrees of visual angle. MoCA: Montreal Cognitive Assessment; HY: Hoehn and Yahr scale; LEDD: Levodopa Equivalent Daily Dose; UPDRS: Unified Parkinson’s disease rating scale. SWJ: Square wave jerks; SWJ is the average percentage of square wave jerks out of all microsaccades. Broken fixation is the average percentage of microsaccades made when fixation was broken out of all microsaccades. * From a total of 128 trials. Missing data is noted as follows. ^a^: average from 10 PD; ^b^: from 11 PD, ^c^: from 12 PD, and ^d^: from 15 PD. The only medication effect difference was in UPDRS (*p* < 0.0001).

## Data Availability

The datasets used and/or analyzed during the current study are available from the corresponding author on reasonable request. The underlying code for this study is not publicly available in its entirety but may be made available to qualified researchers on reasonable request from the corresponding author.

## Supplementary Materials

The following supporting information can be downloaded at: https://www.mdpi.com/article/10.3390/jemr18050046/s1, Figure S1: Distribution of saccade sizes during initial fixation, stimulus, and delay periods for all groups. Distribution is before outlier and square-wave jerk removal; Figure S2: Rate timecourses throughout initial fixation, stimulus presentation, and delay period for (A) Short delay trials only, and (B) Long delay trials only; Figure S3: Rate timecourses (long/short delay combined), not adjusted for baseline rate; Figure S4: Directional Distributions for first and last 500ms of the delay of the OHC, PD ON, and PD OFF, combined across short and long delay trials; Figure S5: Average microsaccade amplitude by target eccentricity (3°, 5°, 7°, 9° of visual angle) for all groups. Error bars represent standard deviation. Amplitudes did not differ significantly within-group by target eccentricity.

## Abbreviations

The following abbreviations are used in this manuscript:

PD	Parkinson’s disease
OHC	Older healthy controls
SWJ	Square wave jerk
