# Supplementary material for: Microsaccade Activity During Visuospatial Working Memory in Early-Stage Parkinson’s Disease
Source: J Eye Mov Res. 2025 Sep 22;18(5):46. doi: 10.3390/jemr18050046 (PMC12565590; doi:10.3390/jemr18050046)
Supplement: Supplementary file 1 [file jemr-18-00046-s001.zip › jemr-3760766-supplementary.pdf]

JEMR-3760766 Supplementary Materials

Supplementary Figure S1

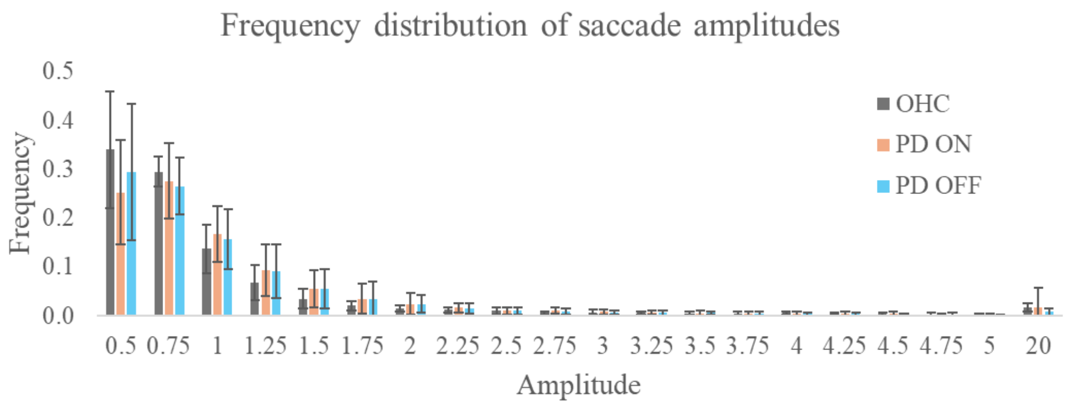

Distribution of saccade sizes during initial fixation, stimulus, and delay periods for all groups. Distribution is before outlier and square-wave jerk removal.

Evaluating short vs long delay type effects on basic characteristics, rate, and directional distributions

Statistical analysis

Two-way mixed/repeated measures ANOVAs were conducted to first assess effects of group/medication (OHC vs PD OFF, OHC vs PD ON, PD ON vs PD OFF) and delay period lengths (short vs long) on basic microsaccade characteristics. These were performed for all microsaccades in the delay period as well as for only those microsaccades occurring in the first or last 500ms of each trial's delay period.

Similarly, mixed-effect 2-way ANOVAs (group x delay type) were used to assess effects of delay types on average rate (separate ANOVAs for rate of the entire delay period, the first 500ms, the middle 500ms, and the last 500ms of the delay).

Results

Microsaccade amplitude and duration were not affected by delay length (p's> .1). Group by delay type interactions also did not reach statistical significance.

For the delay period (whole delay, early, middle, and late) there were no main effects of delay type (short vs long) on average rate for any time period (p's > .4).

Supplementary Figure S2

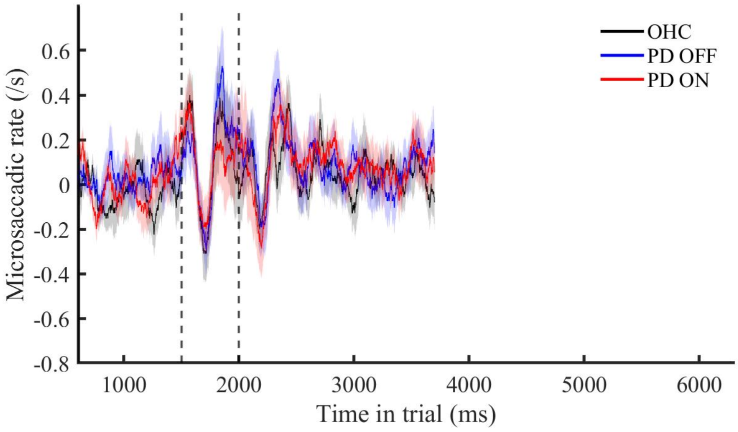

A

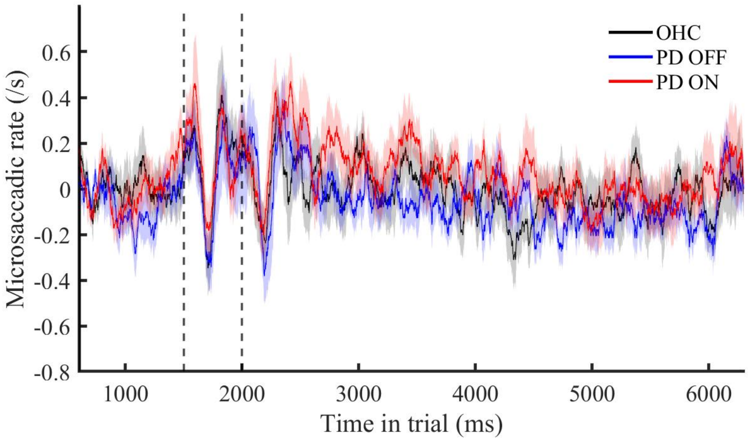

B

Rate timecourses throughout initial fixation, stimulus presentation, and delay period for (A) Short delay trials only, and (B) Long delay trials only.

Supplementary Figure S3

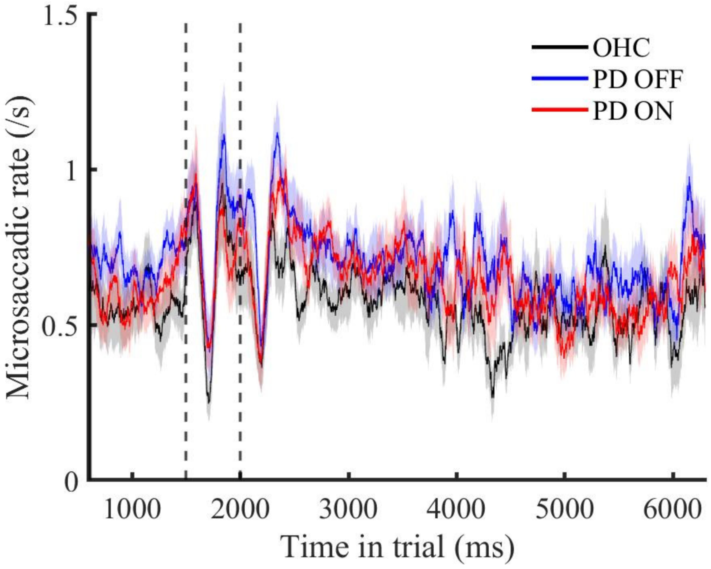

Rate timecourses (long/short delay combined), not adjusted for baseline rate.

Evaluating effect of delay time bin on angular difference from target location

Friedman's tests were conducted to compare within-group directional distributions for the first and last 500ms of delay period microsaccades (short and long delay trials combined). Directional distributions were not affected by time period (p's> .7).

Supplementary Figure 4

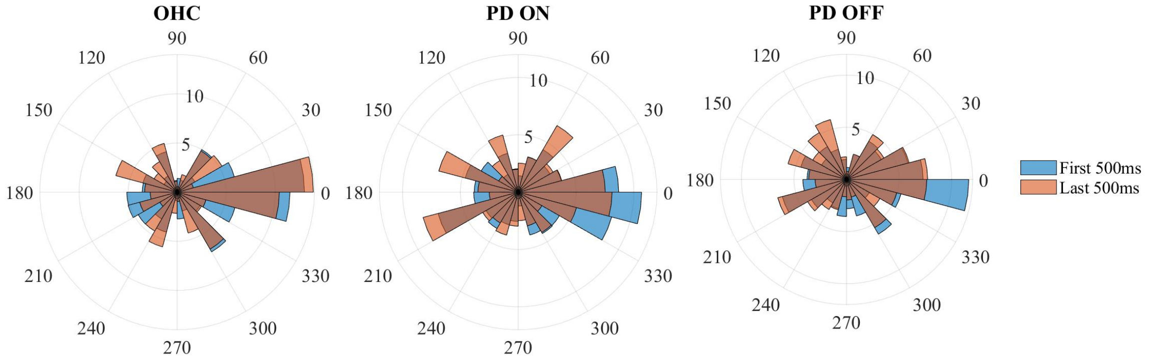

Directional Distributions for first and last 500ms of the delay of the OHC, PD ON, and PD OFF, combined across short and long delay trials.

Supplementary Figure S5

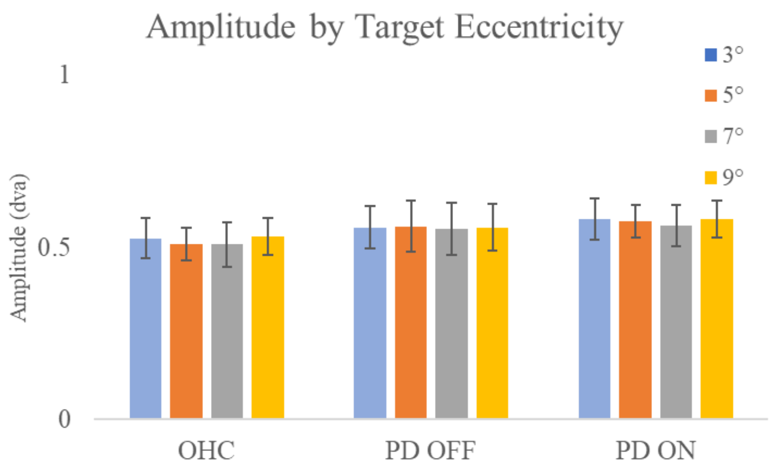

Average microsaccade amplitude by target eccentricity (3°, 5°, 7°, 9° of visual angle) for all groups. Error bars represent standard deviation. Amplitudes did not differ significantly within-group by target eccentricity.
